# Occipital artery-posterior inferior cerebellar artery bypass: a cadaveric feasibility study

**DOI:** 10.1007/s00276-023-03160-5

**Published:** 2023-05-12

**Authors:** Yong Yuan, Xiaolong Wang, Li Han, Yuanzhao Tuo, Bomeng Wu, Xinmin Ding

**Affiliations:** Third Hospital of Shanxi Medical University, Shanxi Bethune Hospital, Shanxi Academy of Medical Sciences, Tongji Shanxi Hospital, Taiyuan, 030032 China

**Keywords:** Bypass, Cadaveric dissection, Complex aneurysm, Occipital artery, Posterior circulation, Posterior inferior cerebellar artery

## Abstract

**Purpose:**

To demonstrate that occipital artery (OA)-p1 posterior inferior cerebellar artery (PICA) bypass can be an alternative for complex posterior circulation aneurysms.

**Methods:**

A far-lateral approach to craniotomy was performed on 20 cadaveric specimens, and the OA was obtained 'in-line.' Its length, diameter, and the number of p1/p2 and p3 segmental perforators were determined, and the relationship between the caudal loop and cerebellar tonsil position was also assessed. The distance between the PICA’s origin and the cranial nerve XI (CN XI), the buffer length above the CN XI after dissection, the OA length required to complete the OA-p1/p3 PICA bypass, and the p1 and p3 segment diameters were all measured. A bypass training practical scale (TSIO) was used to evaluate the quality of the anastomosis.

**Results:**

All specimens underwent OA-p1 PICA end-to-end bypass and had favorable results for the TSIO score, 15 sides underwent OA-p3 PICA end-to-side bypass, and the other bypass protocols were less common. The buffer length above the CN XI after dissection, the distance between the PICA’s origin and the CN XI, and the first perforator were all of sufficient length. The direct length of the OA needed to complete the OA-p1 PICA end-to-end bypass was significantly less than the available length and the OA-p3 PICA end-to-side bypass, with the OA matching the p1 segment diameter. The number of p1 perforators was less than that of p3, and the OA diameter was equal to that of the p1 segment.

**Conclusion:**

OA-p1 PICA end-to-end bypass is a feasible alternative in cases in which p3 segment has high caudal loops or anatomic anomalies.

**Supplementary Information:**

The online version contains supplementary material available at 10.1007/s00276-023-03160-5.

## Introduction

Due to the aneurysm’s deep location, proximity to the brainstem, and a posterior group of cranial nerves, the difficulty in treating complex PICA aneurysms in the posterior circulation, such as aneurysms in the proximal vertebral artery(VA), spindle aneurysms, and dissection aneurysms, is to occlude the aneurysm while ensuring patency of the PICA [14]. Endovascular interventional embolization alone is challenging to densely embolize and has a high recurrence rate. Besides, craniotomy aneurysm clamping alone frequently results in major complications. Therefore, microsurgical occlusion of vertebral artery or PICA aneurysms followed by bypass surgery is a feasible alternative [2, 10, 15, 28]. One of the most common methods for PICA revascularization is OA to PICA bypass [18]. The OA is divided into three distinct sections: ascending cervical, cervical-occipital (or horizontal), and ascending occipital [23]. With an average of 1.9 mm diameter, 79.3 mm length, and 45 mm distance from the external occipital ridge, the horizontal OA is an ideal donor artery for the OA-PICA bypass [4]. The PICA arises from the VA's lateral medullary segment and is distributed around the medulla oblongata and cerebellar earthworms, with variable trajectories, high anatomical variability, and the most complicated relationship with cranial nerves [25]. Based on its course and anatomical relationship with surrounding structures, Lister et al. [20] identified five PICA segments: the anterior medullary segment (p1), the lateral medullary segment (p2), the medullary tonsil segment (p3), the tonsillar segment of the choroid plexus (p4), and the cortical segment (p5). True PICA aneurysms can occur in any of these segments; however, most occur at the VA-PICA junction [16]. Aneurysms in the p4 and p5 segments are classified as distal, while those in the VA-PICA junction and the p1–p3 segments are classified as proximal [2, 24].

**Fig. 1:**
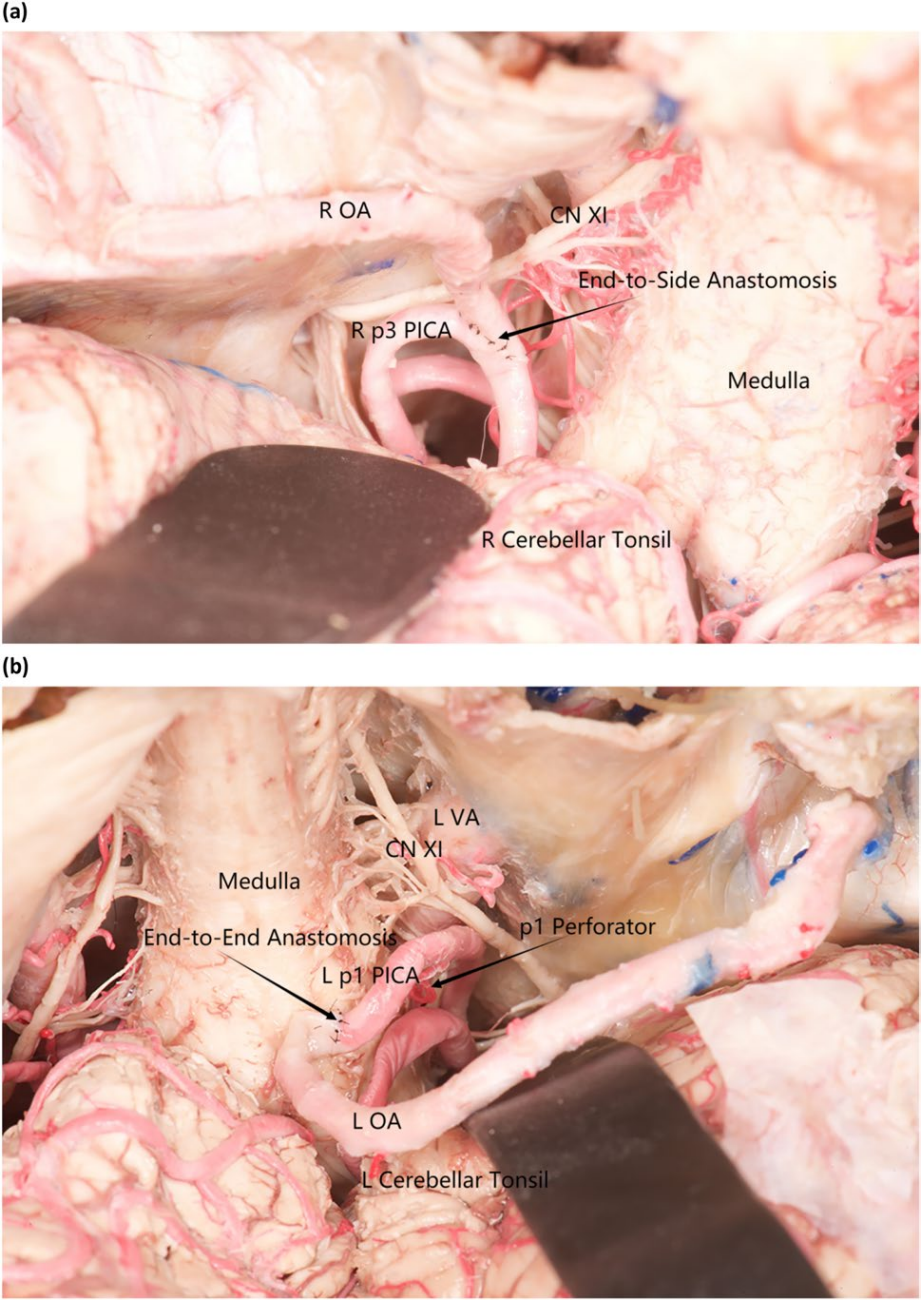
OA is bypassed with p1/p3 PICA, respectively. **a** p3 segment has a significant caudal loop, allowing easy OA-p3 PICA end-to-side bypass anastomosis. **b** p1 segment is cut from the beginning and transposed over CN XI to perform end-to-end anastomosis with OA, which shows a gyrus of the perforators of p1 and a high-riding p3 caudal loop. *L* left, *R* right; *CN* cranial never, *VA* vertebral artery

Due to a large caudal loop and fewer anatomical structures below the tonsillar pole, the OA-PICA bypass site is always located in the tonsillar medullary PICA segment (p3) (Fig. 1a), which is a better choice [1, 27]. However, not all cases have a large caudal loop, and it is challenging to perform direct OA-p3 PICA end-to-side bypass in approximately 28% of cases where the cadual loop is absent or highly located [21]. Therefore, we need to explore other PICA segments as an anastomosis site with the OA through a far-lateral approach in cases where OA-p3 PICA end-to-side bypass is challenging or impossible to complete revascularization. In our literature review, all reported cases were less than 10, the cases were relatively rare, and the time required to collect this series was lengthy. As a result, we used cadaveric head dissection to confirm whether the OA-p1 PICA end-to-end bypass is feasible for this unusual and rare lesion. We dissected the p1 segment from its origin in 10 cadaveric heads (20 occipital arteries in total), transposed it to a more superficial location away from the lower group of cranial nerves, and performed OA-p1 PICA end-to-end bypass (Fig. 1b) to demonstrate its broad applicability for posterior circulation revascularization, particularly as an alternative in complex OA-p3 PICA end-to-side bypass cases.

## Materials and methods

Ten formalin-fixed and embalmed adult cadaveric specimens with cervical tissue (20 sides in total) within 1 year of death were fixed in a prone position on the head frame, with the mastoid process as the highest point of the operative field. We elevated the muscles at the craniocervical junction and used a far-lateral approach with an inverted 'horseshoe-shaped’ skin incision, cutting the skin from the fourth cervical vertebra (C4) upward along the midline to the external occipital ridge, turning outward parallel to the superior collar line to the medial edge of the mastoid process, and downward to 1 cm below the mastoid tip. The severed OA end was at the edge of the transverse incision, which was closed with an arterial clip. The suboccipital muscle and skin were turned outward and pulled with a skin hook to reveal the suboccipital triangle and the source of the mastoid process. The OA was identified at this point along the lateral border of the superior oblique capitis muscle (SOCM), between the SOCM and the posterior belly of the digastric muscle (PBDM), or at the root of the mastoid process, usually covered by the hyaline membrane. The occipital artery was identified intraoperatively via finger perception of fluctuations or ultrasound if embedded in a tendon or thin muscle layer. Once the origin of the horizontal segment of the OA was identified, the OA was obtained and separated from the superior head of the superior oblique tendon and the superficial surface of the cephalic semispinalis muscle in a proximal-to-distal direction to expose the horizontal segment. The OA was extracted from the occipital periosteum, occipital tendon, muscle belly, and soft tissue beneath the capitellum. The donor's vessel was finally completely separated from the tissue to complete the donor vessel acquisition. Neuronavigation was used to measure length and distance, and calipers were used to measure vessel diameter. The diameter across the superior collar line (OA diameter) and the available length of the OA as a donor's vessel (from the bicipital muscle opposite to its superior collar line) were measured.

An occipital craniotomy was performed after scraping the occipital periosteum, the ipsilateral posterior atlantoaxial arch was occluded, and the posterior third of the occipital condyle was removed with a high-speed grinding drill to optimally grind away bone from the lateral margin of the foramen magnum to reveal the vertebral artery entry point. The dura mater was incised in an arc around the occipital condyle, the foramen magnum pool's arachnoid membrane was peeled off under a microscope, and the inferior cerebellar hemisphere was medially retracted to reveal the posterior group of cranial nerves and the PICA. The cerebellum's medullary pool was investigated, segments p1–p5 were dissected and clarified, and the relative position of the cadual loop of segment p3 to the inferior pole of the cerebellar tonsils was observed and recorded. Additionally, the number of perforators emanating from segments p1/p2 and p3 PICA to the brainstem and medulla oblongata was measured. Furthermore, the original distance from the start of the PICA to the CN XI, and to the first perforator (distance from the origin) were measured. The PICA was severed from its origin, and the p1 segment was carefully lifted and pulled out of the posterior group of cranial nerve root gaps and located superficial to the CN XI. The diameter of the p1 segment (p1 diameter) and the buffer length above CN XI (the p1 PICA situated superficial to the CN XI) were measured, without brainstem torsion or excessive distraction. Using 10–0 prolene sutures interrupted by one stitch, two stitches were sewn along the long axis of the vessel as the traction line, four stitches were sewn at equidistant positions between the two traction lines, totaling 10 stitches(Supplement 1). End-to-end anastomosis was performed with the OA on the premise of perforators, and the length of OA required for OA-p1 PICA end-to-end bypass (from the diastasis to the anastomosis of the p1 segment folding over the superficial surface of the CN XI) was measured (OA-p1 PICA end-to-end distance). The ease of the procedure (whether it was necessary to retract the OA) was compared by simulating end-to-side anastomosis of the OA with the PICA p2, p3 caudal loop, p4, and p5 segments. In the OA-p3 PICA end-to-side bypass, the OA length (from the diastasis to the p3 caudal collar anastomosis) and p3 diameter at the anastomosis were measured. Following completion of these measurements, the anastomosis quality was assessed using the TSIO scale [11]: the time required to complete the bypass was recorded by surgical video; the uniformity of the anastomotic suture distribution was observed; the vessel was cut at the occipital artery near the anastomosis, the suture was observed for puncturing the intima of the vessel (whether the intima was intact); finally, signs of stenosis in the anastomosis were to be observed(compare the diameter of the anastomosis with that of the recipient's vessel).

The experimental results were presented as mean and standard deviation. The measured data were generated using SPSS 20.0 software and statistical analysis with the statistical significance set at *P* < 0.05.

## Results

All 20 cadaveric specimens successfully collected OA, and all underwent OA-p1 PICA end-to-end anastomosis without intraoperative cerebellar tonsillar retraction. However, 15 of the 20 specimens had completed OA-p3 PICA end-to-side anastomosis because 5 lacked the caudal loop of the p3 segment. The position of the pontine body had a significant impact on the OA-p3 PICA end-to-side bypass ease (six cases with the cadual loop below the inferior pole of the cerebellar tonsils, six along the inferior pole of the cerebellar tonsils, and three above the inferior pole of the cerebellar tonsils). On the other hand, the remaining segments were bypassed: the OA-p2 PICA, five cases; the OA-p5 PICA, four cases; and the OA-p4 PICA, one case. The cerebellar tonsils were trimmed with the OA in the p3 segment. The donor's vessel was 8.24 ± 1.65 cm in length (range 5.1–12.6 cm), with a mean diameter of 1.65 ± 0.23 mm at the trans-superior collateral line (range 1.3–2.1 mm). The mean original distance from the beginning of the PICA to the CN XI was 12.34 ± 6.69 mm (range 4.4–35.2 mm), and the mean distance from the origin after dissociation and retraction over the CN XI was 10.90 ± 5.58 mm (range 4.3–29.2 mm). In addition, the mean distance to the first perforator was 6.80 ± 2.43 mm (range 2.5–10.5 mm). The p1 segment's mean diameter was 1.74 ± 0.25 mm (range 1.3–2.1 mm). The median number of p1 perforators was 1 (range 0–2), and the OA length required to complete the OA-p1 PICA end-to-end bypass was 3.12 ± 0.57 cm (range 2.0–3.9 cm). The mean diameter of the p3 segment was 1.63 ± 0.25 mm (range 1.3–2.1 mm), and the median number of p3 segmental perforators was 4 (range 1–6), which was significantly higher than that of the p1 segment. The median distance required for the bypass of the OA from the occipital sulcus to the CN XI above the end of the p1 segment was shorter than the median distance required for the bypass to the cadual loop of the p3 segment (3.12 vs. 4.89 cm; *P* < 0.05).All specimens had favorable TSIO scores (range 3–4 points).

## Discussion

Posterior circulation aneurysms comprise 3.8–15% of all intracranial aneurysms [7]. Posterior circulation aneurysms are more likely to rupture than the anterior [9]. The poor prognosis of patients with posterior circulation aneurysms is due to their proximity to the hypothalamus, brainstem, and cranial nerves, which are more prone to giant or spindle-shaped aneurysms and an increased incidence of ischemic effects, local mass effects, and lower group cranial nerve dysfunction [32]. Consequently, treating posterior circulation aneurysms is exceptionally challenging. Cerebral revascularization in the posterior circulation is an important approach for treating aneurysms at PICA origin. The lower third of posterior circulation aneurysms are more amenable to surgical occlusion because complex aneurysms are more prone to surgical exposure and hemodynamic reconstruction, according to the Swiss SOS group [22]. Some neurosurgeons advise against surgical occlusion of large intracranial arteries [30]. In large, giant, spindle-shaped, calcified aneurysms, intraluminal thrombotic aneurysms, or branches originating from the aneurysm wall, some studies advocate cerebral revascularization [34]. There are two types of bypass options for revascularization: intracranial-intracranial (IC-IC) and extracranial-intracranial (EC-IC) bypass [8]. The former consists primarily of PICA-PICA bypass and VA-PICA bypass, while the latter consists primarily of OA-PICA bypass [26]. We always recommend IC-IC bypass over EC-IC bypass for PICA aneurysms or VA dissection aneurysms requiring revascularization because the former has a lower incidence of cerebrospinal fluid leakage and skin complications and may be the best option for patients with some systemic diseases [29, 33]. The PICA-PICA bypass has been used for the revascularization of the PICA. The proximity and parallelism of the bilateral caudal loops of PICA and the relatively uniform caliber of the tonsil’s medullary segment allow for lateral anastomosis. However, it imposes stringent requirements to complete this bypass procedure [5, 19]. Another disadvantage of the PICA-PICA bypass is the possibility of damaging the contralateral PICA. In addition, prolonged clamping or bypass occlusion may endanger the bilateral PICA, resulting in irreversible ischemic complications in the bilateral cerebellum [13]. Furthermore, the PICA-PICA bypass has a disadvantage in that it requires an entirely different anastomotic procedure than the superficial temporal artery (STA)-middle cerebral artery (MCA) bypass, which is unfamiliar to most neurosurgeons [19]. The technique also necessitates suturing in a deeper lumen, making this anastomosis technically challenging [2]. Alternatively, some surgeons perform PICA revascularization using VA-PICA transposition [6]. However, because the brainstem perforators originate from the PICA proximal, moving the PICA is extremely difficult, and performing a VA-PICA anastomosis in a deep and narrow operative area can be equally complex and challenging [31]. Another method of revascularizing the PICA is to insert a graft vessel between the VA and the PICA, such as the VA-radial artery-PICA or the VA-superficial temporal artery-PICA [12]. On the other hand, a double anastomosis increases stenosis risk and technical difficulties [31].

Despite potential complications, the OA-PICA end-to-end bypass is a common treatment approach due to its versatility [33]. Except for OA absence, it can be applied to almost all non-cystic PICA aneurysms. While there are many anatomical variants of PICA, the OA-PICA bypass is independent of the PICA course. Thus, it can be applied in almost all cases. In fact, OA-PICA bypasses outnumber PICA-PICA bypasses in published cases. OA allows adequate extracranial blood flow to the lesioned receptor area, preventing vasospasm and ischemic complications. Furthermore, neurosurgeons are familiar with this end-to-end anastomosis because it is a variation of the well-known STA-MCA. Consequently, OA-PICA bypasses are typically performed easily and with shorter clamping times. Furthermore, unlike the PICA-PICA bypass, this bypass only requires a single PICA. Ischemic complications are restricted to the ipsilateral PICA in bypass occlusion cases [29]. OA-p3 PICA end-to-side bypass is relatively simple for most PICA with a cadual loop; however, PICA has a high potential for anatomic variation, and there is still a proportion of PICA with a missing or highly positioned cadual loop, making OA-p3 PICA end-to-side bypass extremely difficult, if not impossible. As a result, a viable alternative must be investigated. We compared different segmental bypasses of OA and PICA in this cadaveric autopsy trial. The OA-p1 PICA end-to-end bypass demonstrated a wide range of adaptability and was a good alternative, especially when the OA-p3 PICA end-to-side bypass was challenging to perform.

### The OA-p1 PICA end-to-end bypass’s feasibility

The average diameter of the OA suboccipital segment was 1.65 mm in our study, the average available length was 8.24 cm, and the average diameter at PICA origin was 1.74 mm. Owing to its relatively long available distance and close caliber to the recipient vessel (1.65 mm vs. 1.74 mm), OA is an ideal donor artery for OA-PICA bypass. Second, the p1 segment has 1–2 perforators, and the average original distance of p1 along the VA is 12.34 mm. Direct OA-p1 PICA end-to-end bypass is complex due to the PICA's origin in a deep and narrow space, which is challenging to perform and may result in severe injury. Nonetheless, we can cut the PICA from the beginning of the vertebral artery and pull it to the CN XI. The average buffer length on the surface of the CN XI is also 10.90 mm, indicating that the location is relatively superficial, the space is relatively larger, and the field of view is wider, allowing bypass surgery to be performed in a shallow and wide surgical area. This makes the operation easier and much safer, indicating the theoretical feasibility of OA-p1 PICA end-to-end bypass surgery. In practice, we discovered that OA-p1 PICA end-to-end bypass was successfully performed and the anastomosis quality assessment was favorable (Table 1) in all 20 trial groups, particularly in the five groups where OA-p3 PICA end-to-side bypass could not be performed. This demonstrates that the OA-p1 PICA end-to-end bypass may be feasible.

**Table 1:** The score results of OA-p1 PICA end-to-end bypass

Specicmens	Closure time for 10 stitches in 1 mm vessel	Good distribution of stitches	Thread hidden (intima-intima contact)	Size of orifice (equal or wider than diameter of vessel)	Score according to the practical scale (TSIO^a^)
1L	1	1	1	1	4
1R	1	1	0	1	3
2L	1	1	0	1	3
2R	1	1	1	1	4
3L	1	1	0	1	3
3R	1	1	1	1	4
4L	1	1	0	1	3
4R	1	1	1	1	4
5L	1	1	1	1	4
5R	1	1	1	1	4
6L	1	1	1	1	4
6R	1	1	1	1	4
7L	1	1	1	1	4
7R	1	1	1	1	4
8L	1	1	0	1	3
8R	1	1	1	1	4
9L	1	1	1	1	4
9R	1	1	0	1	3
10L	1	1	1	1	4
10R	1	1	0	1	3

^a^TSIO means: time, stitch, intima, orifice. This practical scale (TSIO) was used to evaluate the quality during bypass training and was divided into four evaluation indexes:closure time < 20 min, good distribution of stitches, intima-intima contact(no suture was visible in the vascular lumen), orifice equal or wider than the diameter of the vessel, 1 point for each item that met the requirements, otherwise, 0 points. The final scores were added: 3–4 points were considered favorable [11]

### Advantages of OA-p1 PICA end-to-end bypass

Table 2 results confirm that OA-p1 PICA end-to-end bypass is always possible (100%) and that cutting PICA from the starting point improves the success and ease of OA-p1 PICA end-to-end bypass revascularization. The caliber matching of OA and PICA and the length of the OA are critical factors in the OA-PICA bypass. We know that matched blood calibers provide a stable and efficient blood supply [5]. Lister et al. observed that the PICA diameter at the origin is approximately 2.0 mm [20]. Alvernia et al. discovered that the average OA diameter at the occipital sulcus is approximately 1.9 mm [3], despite having 1.4 mm where it crosses the superior cervical line. In this study, the average diameter of the occipital artery at the superior collar line was 1.65 mm, while the PICA diameter at the start was 1.74 mm, indicating a high caliber match between the recipient and donor vessels of the p1 segment. Second, the OA’s available length as a donor’s vessel reached 8.24 cm (which could be further distally free due to length requirements), while the direct distance to complete the OA-p1 PICA end-to-end bypass was only 3.12 cm, confirming the caliber match and abundant length of the OA-p1 PICA end-to-end bypass. After dissociating the PICA from the origin, its available length over the CN XI reached 10.9 mm. Its location was significantly more superficial (mean distance higher than the CN XI), with a more open operating field and no need to retract the tonsils.

**Table 2:** The OA-p1 PICA end-to-end bypass measurement

Specicmens	Original distance with N11 (mm)	Buffer length above N11 (mm)	p1 or p2 perforators	Distance from origin (mm)	Available length (cm)	OA-p1 distance (cm)	p1 diameter (mm)	OA diameter (mm)	End-to-end bypass^a^
1L	9.7	8.9	2	4.1	8.4	3.3	1.7	1.7	Y
1R	4.4	14.8	1	8.3	7.4	2.7	2.0	1.8	Y
2L	9.9	7.0	1	3.2	9.4	3.4	1.7	1.5	Y
2R	8.8	6.2	1	4.7	8.8	3.8	1.3	1.3	Y
3L	13.3	9.4	1	10.5	8.9	2.9	1.5	1.3	Y
3R	12.0	8.0	2	9.8	9.5	2.9	1.4	1.4	Y
4L	11.1	15.3	0	6.8	7.8	3.6	1.9	1.7	Y
4R	35.2	4.3	2	6.8	12.6	2.6	1.6	1.5	Y
5L	15.0	13.8	0	6.6	7.2	3.8	1.5	1.4	Y
5R	7.7	4.3	1	2.5	10.6	3.8	1.5	1.5	Y
6L	13.6	9.0	0	5.2	6.8	3.9	1.9	1.8	Y
6R	7.6	8.8	1	4.9	7.8	3.7	1.5	1.4	Y
7L	15.7	11.2	2	6.2	7.3	2.9	1.8	1.7	Y
7R	13.0	7.7	2	9.7	9.0	2.6	1.6	1.6	Y
8L	13.5	8.0	1	5.1	5.1	2.0	2.1	1.9	Y
8R	4.9	11.6	0	10.3	6.5	2.2	1.9	1.9	Y
9L	9.6	13.6	1	5.5	8.2	2.9	1.7	1.6	Y
9R	21.4	29.2	2	9.3	7.6	3.1	2.1	1.9	Y
10L	13.2	9.8	2	7.2	9.4	3.3	2.1	2.1	Y
10R	7.2	17.1	1	9.3	6.5	2.6	2.0	1.8	Y
Mean	12.34	10.90	1.15	6.80	8.24	3.12	1.74	1.65	

^a^All 10 cadaveric specimens (a total of 20 occipital arteries) completed OA-p1 PICA end-to-end bypass

**Fig. 2:**
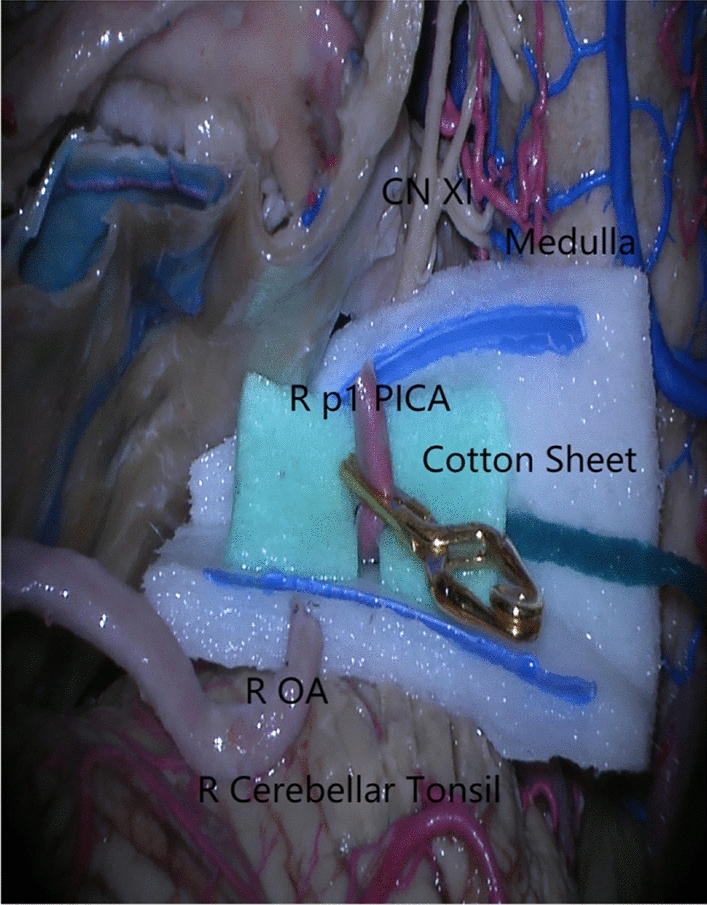
Cotton sheet technique. Stabilizing the p1 PICA stump with a temporary aneurysm clip on a high-gloss heterochromatic cotton sheet prevents excessive tension in the perforator during the bypass procedure. *CN* cranial nerve

When combined with Table 3, the OA-p1 PICA end-to-end bypass outperforms the OA-p3 PICA end-to-side bypass, with fewer perforators and a shorter direct bypass length (*P* < 0.05). The OA-p1 PICA end-to-end bypass, with a median of 11 perforators for p1 or p2 and 44 for p3, poses a lower risk of ischemic injury to the brainstem and medulla oblongata. The average OA length required to complete the OA-p1 PICA end-to-end bypass is 3.12 cm, while the OA-p3 PICA end-to-side bypass requires 4.89 cm. The OA-p1 PICA end-to-end bypass has a shorter anastomotic length, is sutured naturally from below, and can easily rotate to the opposite side. Therefore, the procedure is more efficient and convenient, saving time and reducing ischemic complications. The OA-p3 PICA end-to-side bypass, on the other hand, requires more time and effort to trim the OA to match the p3 segmental arteriotomy. Furthermore, neurosurgeons are more familiar with and technically proficient with OA-p1 PICA end-to-end anastomosis. Nevertheless, the only concern is the high tension of the brainstem perforators. When we elevated the PICA from a lower-positioned starting point to just above the CN XI in this study, the final buffer length above the CN XI was 10.9 mm without tension, which was sufficient for our suture. The p1 segmental dissection was stabilized with an aneurysm clip on a cotton sheet to prevent excessive tension in the perforators (Fig. 2). Finally, the PICA could return to its original position after a successful bypass.

**Table 3:** The others bypass measurement

Specicmens	p3 perforators	OA-p3 distance^a^ (cm)	p3 diameter (mm)	p3 relation with tonsil after dissection^b^	OA-p3^c^ bypass	OA-p5 bypass	OA-p2 bypass	OA-p4 bypass
1L	6	4.7	1.6	Below	+	+ +	−	−
1R	5	No	1.7	No loop	−	+ +	−	−
2L	5	5.5	1.6	Below	+ +	−	−	−
2R	4	4.8	1.3	Below	+	−	−	−
3L	2	4.8	1.4	Along	+ +	−	−	−
3R	1	5.6	1.3	Along	+ +	−	−	−
4L	3	4.9	1.8	Behind	+	−	−	−
4R	6	4.5	1.4	Below	+	+ +	+	−
5L	4	4.4	1.4	Behind	+	−	+ +	−
5R	6	no	1.5	No loop	−	−	+ +	−
6L	3	4.7	1.6	Below	+ +	−	-	+
6R	3	no	1.3	No loop	−	−	+ +	−
7L	5	no	1.6	No loop	−	−	−	−
7R	4	no	1.6	No loop	−	−	−	−
8L	2	4.7	2.0	Along	+ +	+ +	−	−
8R	4	4.8	1.9	Along	+	−	+ +	−
9L	3	5.2	1.6	Behind	+	−	+	−
9R	3	5.0	2.0	Below	+ +	−	+	−
10L	2	4.9	2.1	Along	+ +	−	−	−
10R	5	4.8	1.9	Along	+ +	−	−	−
Mean	3.80	4.89	1.63					

^a^‘No’ means that the bypass cannot be performed without measuring the distance; ^b^5 of the 20 side specimens had no tail rings, and the 6 side specimens were located below the lower tonsil pole, 6 sides at the tonpole level, and 3 sides above the tonsil pole; c-: negative; + : can be done but need retraction; +  + : bypass without retraction

### Other OA-PICA bypass sites

Matsushima et al. demonstrated that OA bypass to the p2-5 segment of PICA is feasible [24]. Other possible bypasses are listed in Table 3. The caudal loop of the p3 PICA was missing in 5 specimens, and only 15 of 20 underwent bypass of the OA-p3 PICA end-to-side anastomosis. The remaining segmental bypasses were carried out as follows: OA-p2 PICA bypass on 5 sides, OA-p5 PICA bypass on 4 sides, and OA-p4 PICA bypass on 11 side. The PICA cadual loop was large and did not require any further dissection or constriction in 8 of the 15 completed OA-p3 PICA end-to-side bypasses; 7 had the PICA ring running along the tonsillar pole or below the tonsils, and performing the OA-p3 PICA end-to-side bypass required retraction of the tonsils to obtain adequate exposure of the cadual loop. The difficulty of the OA-p3 PICA end-to-side bypass was largely determined by the position of the p3 segment in relation to the tonsils. Lister et al. described 23 caudal loops above the tonsillar pole [20], 11 at the tonsillar pole level, and 8 below the tonsillar pole in 42 lateral PICAs, implying that only a few OA-p3 PICA end-to-side bypasses can be easily performed without tonsillar retraction. Most brainstem perforators originate from the tonsilomedullary segment (p3), which limits their movement and puts them at greater risk of injury during manipulation. Furthermore, end-to-side bypass necessitates more suturing and bypass techniques, particularly if the OA lacks a curved length to suture to the tonsillar side, making the operation more challenging.

**Fig. 3:**
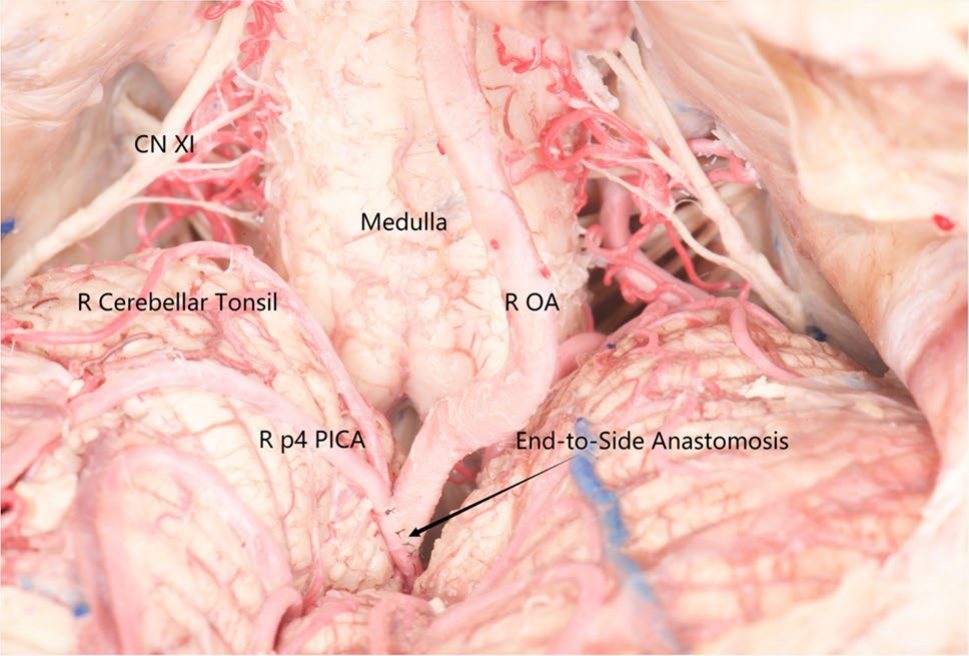
OA-p4 PICA end-to-side bypass. End-to-side bypass anastomosis of OA with p4 PICA was performed, and OA was seen to be almost straightened. *L* left, *R* right, *CN* cranial nerve

OA-p5 PICA end-to-side bypass is another good option; however, it is more challenging due to the p5 segment diameter. In addition, some specimens have a tortuous lateral medullary ring that is easily pulled to a relatively superficial position or a wide gap between both sides of the tonsils; therefore, OA-p4 PICA end-to-side bypass has also been attempted in some specimens (Fig. 3). However, OA-p4, p2, and p5 PICA end-to-side bypasses are more challenging to operate on and are not the preferred option in clinical practice.

### OA-p1 PICA end-to-end bypass clinical situation and limitations

Endovascular coil embolization with stenting is an excellent treatment option for VA dissection aneurysms. Nonetheless, Kim et al. demonstrated that PICA origin is the only independent risk factor for recurrence after endovascular treatment of VA dissection aneurysms because blood flow to the PICA is preserved. They hypothesized that the continuous blood flow through the unprotected residual dissection aneurysm to the PICA was responsible [17]. The OA-p1 PICA end-to-end bypass has the distinct advantage of providing good hemodynamic reconstruction when the aneurysm must be removed by occluding the VA and initiating PICA. As a result, the primary clinical scenario for this technique is VA dissection aneurysms involving PICA and small aneurysms at the VA-PICA junction that cannot be clamped. Therefore, this technique is not appropriate for distal PICA. It depends on how close the aneurysm is to the perforators when determining whether proximal PICA aneurysms need hemodynamic reconstruction. We can still easily pull p1 over the CN XI after dissection from the beginning; however, Table 2 presents one specimen with the p1 segment origin only 2.5 mm from the first perforator. This suggests that OA-p1 PICA end-to-end bypass may damage the perforators, allowing ischemic damage to the brainstem or medulla oblongata. Therefore, in actual clinical cases, if the aneurysm is less than 2.5 mm to the first perforator, an OA-p1 PICA end-to-end bypass may be ineffective. Furthermore, due to the proximity of the PICA initiation requiring dissection to the lower brainstem and lower cranial nerve, the operation location is deep, which is anatomically tricky and may result in medically induced injury during the surgical procedure.

Therefore, we recommend future clinical applications: preoperative angiography, measurement of OA and p1 caliber matching, and available OA length. Another critical consideration is the PICA origin. When the PICA origin is close to or even higher than the confluence of the bilateral VA, it indicates that the PICA origin depth is far from the CN XI. To effectively expose the PICA origin and facilitate bypass, a far-lateral approach is used, abrading the posterior one-third or more of the occipital condyle. The skin incision for OA capture can be straight, 'C-shaped', or 'L-shaped'. However, stripping to the superior cervical line is required. Long, nonlinear skin incisions are unfavorable with regard to flap invasiveness; nevertheless, they are better in terms of surgical access. For instance, the ‘L-shaped’ skin incision and the musculocutaneous flap can be flipped caudally, followed by a large far-lateral craniotomy to take advantage of a wide, gently angled, and relatively shallow surgical field. After the aneurysm has been captured and the PICA and VA have been dissected, the OA-p1 PICA end-to-end bypass will be performed without further dissection. A temporary aneurysm clip is placed on the donor vessel after a high-gloss heterochromatic cotton sheet is set as a background, which can easily stabilize the already separated p1 stump and prevent excessive tension in the perforators during the bypass procedure.

## Conclusion

OA-p1 PICA end-to-end bypass has fewer anatomic structures, more room to operate, good caliber matching, is easy to perform, has a high success rate, and can ensure adequate hemodynamic supply to the PICA supply area, and its anastomosis quality is favorable. It can not only be used as an OA-p3 PICA end-to-side bypass alternative that is difficult to complete but it can also be widely used as a bypass treatment in complex aneurysms in the posterior circulation or VA dissection aneurysms involving the PICA.

## Supplementary Information

Below is the link to the electronic supplementary material.

Supplementary file1 (MP4 254668 KB)

## Data Availability

The authors confirm that the data supporting the findings of this study are available within the article and its supplementary material.
